# Plastic-film mulching and urea types affect soil CO_2_ emissions and grain yield in spring maize on the Loess Plateau, China

**DOI:** 10.1038/srep28150

**Published:** 2016-06-22

**Authors:** Qiaofei Liu, Yu Chen, Weiwei Li, Yang Liu, Juan Han, Xiaoxia Wen, Yuncheng Liao

**Affiliations:** 1 College of Agronomy, Northwest A&F University, Yangling 712100, China

## Abstract

A 2-year field experiment was conducted on maize (*Zea mays* L.) to explore effective ways to decrease soil CO_2_ emissions and increase grain yield. Treatments established were: (1) no mulching with urea, (2) no mulching with controlled release fertiliser (CRF), (3) transparent plastic-film mulching (PMt) with urea, (4) PMt with CRF, (5) black plastic-film mulching (PMb) with urea, and (6) PMb with CRF. During the early growth stages, soil CO_2_ emissions were noted as PMt > PMb > no mulching, and this order was reversed in the late growth stages. This trend was the result of topsoil temperature dynamics. There were no significant correlations noted between soil CO_2_ emissions and soil temperature and moisture. Cumulative soil CO_2_ emissions were higher for the PMt than for the PMb, and grain yield was higher for the PMb treatments than for the PMt or no mulching treatments. The CRF produced higher grain yield and inhibited soil CO_2_ emissions. Soil CO_2_ emissions per unit grain yield were lower for the BC treatment than for the other treatments. In conclusion, the use of black plastic-film mulching and controlled release fertiliser not only increased maize yield, but also reduced soil CO_2_ emissions.

Climate change, caused primarily by increased concentrations of CO_2_ in the atmosphere, and food security problems, due to the fast-growing human population and loss of farmland, have become global issues that seriously threaten developing countries^1^. Agriculture is a source of CO_2_ emissions, with its annual contribution to climate change approximately 14%^2^. Small changes in the amounts of soil CO_2_ emissions could have a large effect on the concentration of CO_2_ in the atmosphere^3^. To mitigate the potential negative effects of climate change on ecosystems and human well-being, a series of strategies are needed to reduce CO_2_ emissions and atmospheric CO_2_ concentrations^3^,^4^.

Spring maize (*Zea mays* L.) is one of the most popular grain crops in the semiarid Loess Plateau region, but low air temperatures and drought in April and May often results in poor plant establishment. In dryland farming systems, plastic-film mulching has been used for the micro-catchment of water and to increase topsoil temperature for many years^5^. This technique can improve grain yields and water use efficiency in rain-fed regions^6^ and has now been widely applied to maize, wheat, cotton, and potato in semiarid regions^5^,^6^,^7^.

The emission of soil CO_2_ is complex and variable, and is controlled by many abiotic and biotic factors^1^. Soil CO_2_ emission involves organisms metabolizing substrates that produce CO_2_ within the soil matrix^8^, the microbial decomposition of organic matter (heterotrophic respiration), and root respiration (autotrophic respiration). It ultimately results in the movement of CO_2_ through soil pores, and the release from the soil system can be measured at the soil surface^8^. Soil CO_2_ emission in agro-ecosystems is highly sensitive to management practices. Field management practices, such as land use, tillage, fertilisation, and cropping practices, significantly affect CO_2_ emissions from cropland^4^. In terms of reducing the effect of agriculture on climate change, the objective of sustainable agriculture is to increase grain yield and decrease soil CO_2_ emissions. Our previous study indicated that plastic-film mulching combined with controlled release fertilisers could increase the grain and biomass yield of maize^7^. However, the effects of mulching on soil CO_2_ emissions are variable. In a study in the north China Plain, soil CO_2_ emissions from a maize field in 2012 and 2013 were 35.4% and 19.9% lower, respectively, for the mulching treatments than for the non-mulching treatments^1^. However, in a spring maize field study, soil CO_2_ emissions were higher in the mulching treatment than in the no mulching treatment, with even greater emissions in the ‘mulching + N fertiliser’ treatments^9^.

Urea is the most widely used fertiliser globally because of its high nitrogen content (46%), low cost, and ease of application^10^. When applied to the soil, urea undergoes a series of biological, chemical and physical transformations to produce plant nutrients^11^. Since plants need only a small quantity of food during early growth, excess nutrients are lost due to leaching through hazardous gaseous emissions^12^. Controlled release fertilisers (CRFs), which provide a gradual nutrient supply for a long period, may overcome the problems^13^,^14^. CRFs are made of soluble fertilisers that are coated with materials, such as sulphur, polymer, and other synthetic substances, that delay the release of the soluble fertiliser^13^. These products have been successfully used to limit nutrient losses to the environment^14^. However, CRFs tend to be more expensive than conventional fertilisers, can have unpredictable nutrient release, and some coating materials can even harm the environment^15^. Sulphur was initially used for urea coating as it is cheap, has fungicidal properties, is biodegradable, and also acts as a secondary plant nutrient which may promoting the development for crop^15^,^16^. However, the sulphur coating shell is fragile and can be ineffective at controlling nutrition release. Polymer-coated CRFs overcome the shortages of sulphur-coated CRFs, and provide a supply of nutrients over longer periods of time, consistent with crop metabolic needs. However, not only are polymer-coated fertilisers more expensive, but they can also release hazardous emissions into the environment^15^. Wu *et al*.^17^ reported that thicker coating layers may damage soil quality if they are not degraded in parallel with nutrient release. Detrick *et al*.^18^ reported that since sulphur shells left in the soil are not immediately integrated, an excessive amount of sulphur may build up and react with water to acidify the soil. Overall, all these issues limit the use of CRFs for field crops^15^. Therefore, low cost, easily fabricated, and environmentally friendly CRFs are urgently needed.

Previous studies on plastic-film mulching have mainly focused on transparent plastic-film mulching (PMt)^1^,^3^,^5^,^6^,^9^, while only a few studies on black plastic-film mulching (PMb) have been conducted^19^,^20^. Information on the combined effects of PMb and CRFs is limited, and therefore the mechanisms underlying the reduction of soil CO_2_ emission through the use of these to tools remain unknown. Therefore, it is necessary to evaluate the effects of management practices on soil CO_2_ emission and grain yield so that alternative farming strategies can be suggested to maize farmers in China. Accordingly, the objectives of this study were to: (1) identify the effects of plastic-film mulching and urea types on soil CO_2_ emissions and maize yield, (2) determine the optimal combination of mulching and urea types to increase maize yield under various tillage systems, and (3) suggest the optimal combination of management practices to reduce soil CO_2_ emission and increase maize yield in the semiarid Loess Plateau region of China.

## Results

### Weather conditions

**Figure 1:**
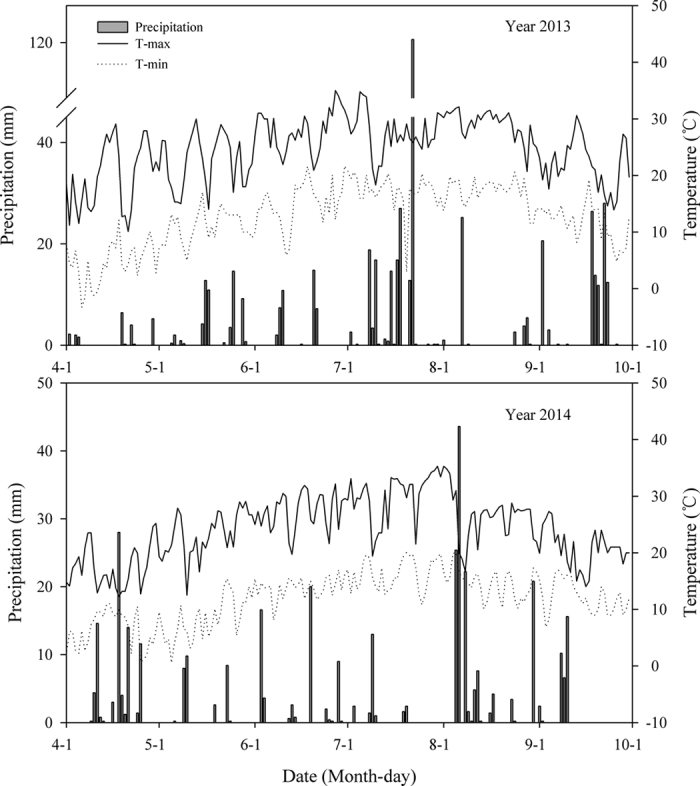
Daily minimum and maximum temperatures (°C) and rainfall (mm) at the experimental site throughout the study period (2013 and 2014).

The daily minimum and maximum air temperatures and precipitation recorded during the two-year experiment are shown in Fig. 1. The trends for both temperature and rainfall were considerably different between experimental years. In general, the maize growing season was hotter and drier in 2013 than in 2014. Precipitation during the spring growing season accounted for 87.6% (513.2 mm) and 75.8% (615.6 mm) of annual precipitation in 2013 and 2014, respectively. However, precipitation varied from season to season. For example, precipitation was higher in July 2013 than in July 2014 and considerably lower in June, August, and September 2013 than in June, August, and September 2014.

The minimum and maximum air temperatures also varied greatly between the two experimental seasons (Fig. 1). Lower temperatures were reported in July 2013 than in July 2014, while higher temperatures were noted in August 2013 than in August 2014. The differences in rainfall and air temperature between the experimental seasons were expected to affect maize development and cause crop yield variations.

### Soil temperature and moisture

**Figure 2:**
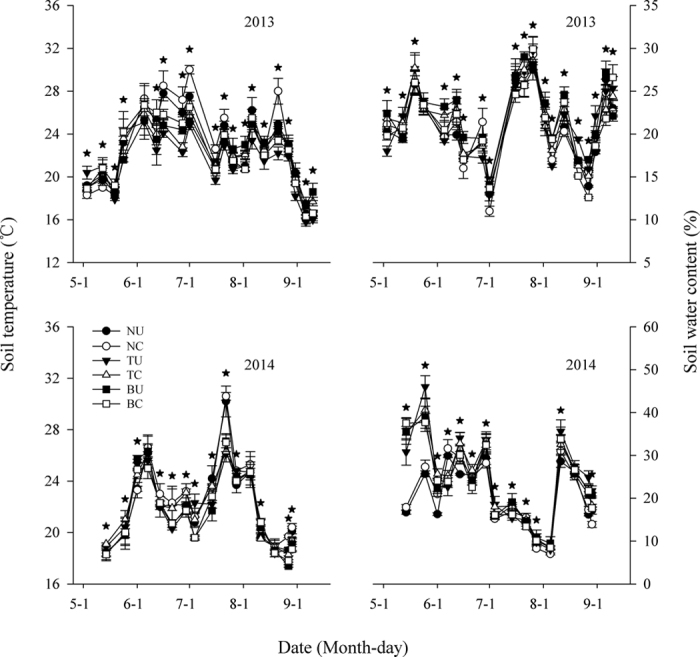
Soil temperature and moisture at 0–5 cm depth under various treatments in two maize growing seasons. Stars show LSD_0.05_ values. Bars show means ± s.e.m. NU, no plastic-film mulching with urea; NC, no plastic-film mulching with controlled release fertiliser (CRF); TU, transparent plastic-film mulching (PMt) with urea; TC, PMt with CRF; BU, black plastic-film mulching (PMb) with urea; BC, PMb with CRF.

Soil temperature was strongly related to the weather conditions observed during the study period (Fig. 2). The highest soil temperature values were reached on 1 July 2013 and 22 July 2014 (26.6 and 27.9 °C, means of all treatments, respectively), whereas the lowest values were observed on 6 September 2013 and 27 August 2014 (16.6 and 18.4 °C, means of all treatments, respectively). In both experimental years, before June, soil temperature was higher in the mulch treatments than in the no mulch treatments, whereas after June, the soil temperature tended to be higher under the no mulch treatment because of maize plant shading.

In general, in both years, soil moisture depended on weather conditions (Fig. 2). As expected, the dynamics of soil moisture were the reverse of soil temperature. The soil water content was higher in the mulching treatments than in the no mulching treatments. In 2013, the soil water content was often higher in the black plastic-mulch treatments than in the transparent plastic-mulch treatments, whereas the opposite was noted in 2014.

### Soil CO_2_ emissions

**Figure 3:**
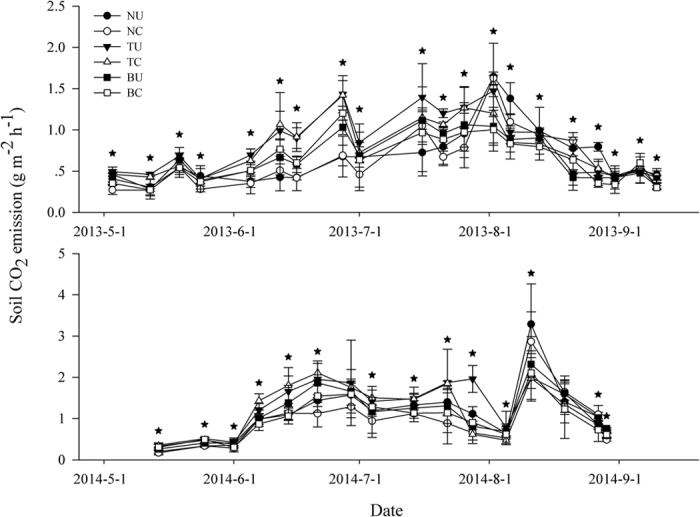
Soil CO_2_ emissions under various treatments in two maize growing seasons. Stars show LSD_0.05_ values. Bars show means ± s.e.m. NU, no plastic-film mulching with urea; NC, no plastic-film mulching with controlled release fertiliser (CRF); TU, transparent plastic-film mulching (PMt) with urea; TC, PMt with CRF; BU, black plastic-film mulching (PMb) with urea; BC, PMb with CRF.

In both experimental years, the trends for soil CO_2_ emissions were similar throughout the study period, with gradually increasing levels in August and sharply decreasing levels in September (Fig. 3). In general, soil CO_2_ emissions were lower in 2013 than in 2014. From May to August in 2013, soil CO_2_ emissions gradually increased for all treatments, with high levels for the transparent plastic mulching (PMt) with urea (TU) and the PMt with CRFs (TC) treatments, intermediate levels for the black plastic mulching (PMb) with urea (BU) and PMb with CRFs (BC) treatments, and low levels in the no mulching with urea (NU) and the no mulching with CRFs (NC) treatments. In contrast, after early August, soil CO_2_ emissions tended to be higher in the NU and NC treatments and lower in the BU and BC treatments. In 2014, the first peak of soil CO_2_ emissions for all treatments occurred in late June, corresponding to the increase in air temperature during the same period. However, in July, soil CO_2_ emissions tended to decrease for all treatments, even though air temperatures remained high and rainfall was absent. After the second peak point was reached in mid-August, soil CO_2_ emissions for all treatments decreased sharply until September. Similar to 2013, soil CO_2_ emissions in 2014 were initially higher for TU and TC, intermediate for BU and BC, and lower for NU and NC, whereas after mid-August, soil CO_2_ emission tended to be higher for NU and NC and lower for the treatments with mulching.

Table 1 shows the results of the two-way ANOVA for soil CO_2_ emissions (g m^−2^ h^−1^) for mulching (M), urea types (U), and the M × U interaction during the seedling (5 June 2013; 1 June 2014), jointing (1 July 2013; 4 July 2014), flare opening (26 July 2013; 28 July 2014), blooming (6 August 2013; 5 August 2014), filling (13 August 2013; 11 August 2014), and milk (31 August 2013; 29 August 2014) stages of maize. For both years, plastic-film mulching significantly affected soil CO_2_ emissions in all the growing stages (P ≤ 0.05), except for the filling and milking stages in 2013 (P ≥ 0.05). Urea types only significantly affected soil CO_2_ emissions during the flare opening (P ≤ 0.01) and blooming stages (P ≤ 0.01) in 2013 and 2014, and during the seedling stage in 2014 (P ≤ 0.05). The interaction effect of M × U on soil CO_2_ emissions was varied, but there was a significant effect during the flare opening and milk stages in 2013(P ≤ 0.05), and the seedling, flare opening, and blooming stages in 2014 (P ≤ 0.05).

### The relationship between soil CO_2_ emissions and soil temperature and moisture

Table 2 shows the correlations between soil CO_2_ emissions (F), soil temperature (T) and soil water content (W) at a 5-cm depth under NU, NC, TU, TC, BU and BC in 2013 and 2014. None of the R^2^ were significant (P > 0.05), except for the correlation of soil CO_2_ emission with soil water content in the NC treatment in 2014 (R^2^ = 0.501, P < 0.05). In general (excluding this one correlation), there was no correlation between soil CO_2_ emissions and soil temperature and soil water content at a 5 cm depth or at other soil depths (data not shown).

### Cumulative soil CO_2_ emissions and yield

Mulching and urea types significantly affected the cumulative soil CO_2_ emissions in both years (P < 0.01) and there was significant variation in the interaction effect (M × U) between the two years (P < 0.05; Table 3). In 2013 and 2014, cumulative soil CO_2_ emissions were 22.0% and 16.% higher in the TU treatment than in the NU (control) treatment, respectively (Table 4). In both years, there was no significant difference between the BU and NU treatments for cumulative soil CO_2_ emissions (P > 0.5; Table 4).

For grain yield, significant variation was noted for the mulching and urea treatments, and the M × U interaction (P < 0.05; Table 3). The highest grain yield was recorded in the TC treatment in 2013 (mean ± s.e.m.: 12.35 ± 1.12 t ha^−1^) and in the BC treatment in 2014 (16.64 ± 1.49 t ha^−1^; Table 4). Overall, grain yields were higher in 2014 than in 2013 (Table 4).

Mulching and urea types significantly affected the cumulative soil CO_2_ emissions per unit grain yield in both years, and there was significant variation for the M × U interaction (P < 0.05; Table 3). The highest soil CO_2_ emissions per unit grain yield were noted for the TU treatment (2.27 ± 0.13 in 2013 and 2.33 ± 0.07 in 2014), while the lowest were noted for the BC treatment (1.54 ± 0.14 in 2013 and 1.59 ± 0.09 in 2014; Table 4). The cumulative soil CO_2_ emissions per unit grain yield were significantly lower (27.1% in 2013 and 29.3% in 2014) in the BC treatment than in the NU treatment (P < 0.05; Table 4). Overall, higher amounts of soil CO_2_ emissions per unit grain yield were noted for the current management practice treatments (transparent plastic-film mulching and urea) than the alternative management practice treatments (black plastic-film mulching and CRFs).

## Discussion

In the two early growth stages of maize, the soil CO_2_ emissions were in the following order (highest to lowest): PMt > PMb > no mulching. The higher soil CO_2_ emission in the mulching treatments is consistent with the results of previous studies^9^. In contrast, in the late growing stage of maize, the order of soil CO_2_ emissions was reversed: no mulching > PMt > PMb. These findings are consistent with those of Jing^18^, but opposite to the findings of Chen *et al*.^21^ and Xin *et al*.^20^. This reversal trend is caused by the following factors. First, because of shading, the warming effect of plastic-film mulching becomes weaker with on-going plant growth. Second, plastic-film mulching can maintain higher soil water content, which can partially inhibit soil CO_2_ emissions. Finally, plastic-film mulching prevents air interchange between the soil and atmosphere, and therefore the accumulation of soil CO_2_ under the plastic film could inhibit the release of soil CO_2_. These dynamics of soil CO_2_ emissions caused the cumulative soil CO_2_ emission to be higher in the PMt treatments and lower in the PMb in both experimental years (Table 4). The lowest cumulative soil CO_2_ emission was observed in the BC treatment in both years (Table 4).

Many previous studies have found soil temperature to be the key factor in soil CO_2_ emission dynamics, because it controls the activity of the soil biological community, plant growth, and soil hydrological processes^22^. In this study, early in the growing season, soil temperature was higher in the mulching treatments than in the non-mulching treatments. In 2013, soil temperature (depth of 5 cm) was often higher in the PMt treatments than in the PMb treatments. In contrast, in 2014, the order was PMb > PMt > no mulching. Late in the growing season, soil temperature was often higher in the no mulching treatments than in the mulching treatments, and this was likely due to plant shading. Li *et al*.^9^ noted that plastic-film mulching could increase the soil temperature of spring wheat in the 5 cm layer, and the changes showed a ‘U’ pattern, that is, the effect was significant during the early and late growth stages and insignificant during the middle growth stage. Jing^19^ studied the effect of PMt and PMb on a poplar forest and found that PMt had a greater warming effect than PMb, but the warming effect weakened with increasing soil depth. However, Xin *et al*.^20^ explored the effects of PMt and PMb on an apricot orchard and found that soil temperature was 1.4 °C higher with PMb than with PMt. Other studies also found that film mulching could increase soil temperature^23^,^24^,^25^. The reason for the warming effect may be attributed to solar radiation, which is a unique source for soil surface heat. Plastic film can absorb solar energy and store it in the soil. The thermal radiation ratios of transparent and black plastic film are 80–90% and 30–40%, respectively. Thus, transparent plastic film can take in more solar energy than black plastic film and make the topsoil temperature higher^26^. Moreover, black plastic film warms quickly under sunshine, but delivers less energy to the soil because it is less transparent, and therefore it has less of a warming effect.

Soil moisture is also a major factor affecting soil CO_2_ emission in semi-arid regions^27^,^28^. In general, in both experimental years of our study, the soil water content was higher in the plastic-film mulching treatments than in the non-mulching treatments. In 2013, soil water content in a few treatments was noted as PMb > PMt > no mulching. However, in 2014, soil water content under all treatments was noted as PMb > PMt > no mulching. Thus, in this study, more soil water could be retained with PMb than with PMt, which is consistent with the results of Xin *et al*.^20^. However, Jing^19^ studied the effect of plastic-film mulching on a poplar forest and found that film mulching could increase soil water content, but found no significant difference between PMt and PMb. Li *et al*.^9^ designed a two-year experiment on spring wheat and found that plastic-film mulching had almost no effect on the soil water storage amount in the top 2 m of the soil, but film mulching could significantly increase the soil water content in the 0–20 cm layer. The reason was that plastic-film mulch could prevent soil water evaporation and store water in the soil. Black plastic-film mulch could prevent water drops from moving upward and evaporation from the sides because it is less transparent and has a weaker warming effect on soil temperature; hence, black plastic-film mulch could store more water in the soil than transparent plastic-film mulch^26^. In addition, Fan *et al*.^23^ noted in a maize experiment that plastic-film mulch could increase topsoil water content and that the greater the mulching rate, the more soil water is stored. Zhang *et al*.^25^ found that for a millet crop, soil water content (depth of 0–10 cm) was 1.25–3.66% higher in the plastic-film mulch treatment than in the control setup.

Previous studies have shown that soil CO_2_ emissions vary significantly with soil temperature^29^,^30^,^31^,^32^, and response mechanisms to soil moisture are extremely complex^32^. Zhang *et al*.^33^ showed in their sites that soil respiration increased exponentially with soil temperature and was significantly influenced by soil moisture, except in woodland and cropland at relatively low temperatures. However, in this current study, there were no significant correlations between soil CO_2_ emissions and soil temperature and moisture. This suggests that other factors are involved in determining the quantitative variation in soil CO_2_ emissions^34^,^35^.

In regards to the cumulative soil CO_2_ emissions per unit grain yield, in both experimental years, significantly lower values were noted for the BC treatment than for the other treatments. This result can be explained as follows. First, plastic-film mulching increased grain and biomass yield by promoting suitable soil temperature and moisture conditions. Higher grain yield was noted for the PMb treatments than for the PMt treatments because the PMb treatments stored more soil water^7^. Second, plastic-film mulching inhibited soil CO_2_ emission in the late growing season of maize, and the inhibition effect was greater in the PMb treatment than in the PMt. In addition, urea types significantly affected soil CO_2_ emissions during flare opening and blooming stage, and the cumulative soil CO_2_ emission during the growing seasons. Previous studies have shown that CRF could slow down the release rate of nutrition in fertiliser, to more suitably meet the nitrogen requirements of maize, and thus promote crop growth, increase crop yield, and increase the fertiliser use rate^8^,^36^. In this study, treatments with the CRFs (TC and BC) produced higher grain yield and lower cumulative soil CO_2_ emissions. Many studies also indicate that CRFs are useful for the reduction of N_2_O emissions from fertilised soils^37^,^38^,^39^,^40^. The use of controlled release technologies, by affecting the timing of nitrogen (N) release from fertiliser^41^, has the potential to reduce both leaching losses of NO_3_^−^ and volatile losses of N as NH_3_ and N_2_O emissions. Reductions in these losses may improve N use efficiency and provide greater stability in fertiliser N performance^41^. Some research noted that the coating material of CRFs have some damage to the soil^15^,^17^,^18^. Sulphur as a secondary plant nutrient, may promote the development of crop and suicide insects in the soil because of its fungicidal properties^15^,^16^. However, the possible effects of sulphur and ploymer to maize and the soil have not been measured during this experiment. Thus, further studies are needed to explore the mechanism of CRFs in maize ecosystems and the exact effect of its coating material to environment.

## Materials and Methods

### Study site

The study was conducted in 2013 and 2014 at the Changwu Agriculture Research Centre in the Shaanxi Province of China (107° 40′ E, 35° 12′ N; 1200 m above sea level). The study site is characterized by a continental monsoon climate. The annual mean precipitation (last 50 years) is 581.2 mm, 64.4% of which occurs between June and September. The annual mean air temperature is 9.4 °C, and the ≥10 °C accumulated temperature is 3029 °C. The annual sunshine duration is 2230 h, with annual total radiation of 484 kJ cm^−2^, and a frost-free period of 171 days. The groundwater in the region is unavailable for plant growth. The soil in the top 1.0 m was a silty loam texture (USDA texture classification system), and the mean bulk density was 1.29 g cm^−3^. The available N, P, and K were 37.90, 10.12, and 129.64 mg kg^−1^, respectively. The soil organic matter content, at a depth of 0–20 cm, was 6.92 g kg^−1^ and soil pH was 7.4.

### Experimental design and execution

**Figure 4:**
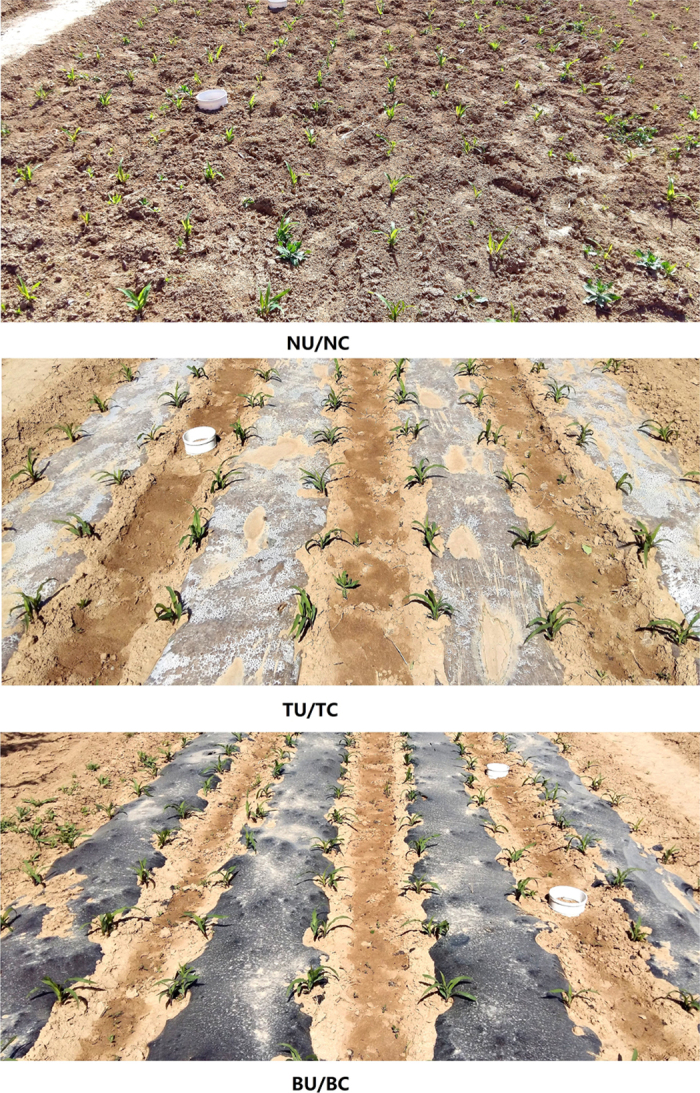
Pictures of the experiment site after maize emergence. The mulching treatments include no mulching (NU/NC), white plastic-film mulching (TU/TC), and black plastic-film mulching (BU/BC).

The experiment was arranged as a randomized block split-plot design (three replicates), with three mulching treatments serving as the main-plot treatments and two urea types serving as the sub-plot treatments (14 m × 3.5 m plots). Thus, six treatments were established: (1) no plastic-film mulching with urea (NU), control treatment; (2) no plastic-film mulching with CRFs (NC); (3) PMt with urea (TU); (4) PMt with CRFs (TC); (5) PMb with urea (BU); and (6) PMb with CRFs (BC; Fig. 4). Volfertile fertiliser (Shandong Kingenta Ecological Engineering Co. Ltd), which contains sulphur as an outer coat (≤4% sulphur content) and polymer as a secondary coat, was used as the CRF in the experiment. The nutrient content of the Volfertile was ≥40%, with a N:P_2_O_5_:K_2_O ratio of 29:5:6. Both urea fertilisers were added at a rate of 225 kg N ha^−1^ at sowing. Transparent/black plastic films (80 cm wide and 0.08 mm thick) were applied to treatments using a no-tillage seeder.

The maize cultivar, Pioneer 335, was sown on 20 April 2013 and 28 April 2014, at a plant density of 62,500 plants ha^−1^. A phosphate and potash fertiliser with a N:P_2_O_5_:K_2_O ratio of 1:0.5:0.6 was applied to all plots at sowing. At maturity (17 September 2013 and 18 September 2014), two 1 m^2^ replicates were harvested from the centre of each plot by cutting the stalk directly at the soil surface. Samples were used to determine grain and biomass yields. The dry weight of straw biomass was obtained after drying (oven at 70 °C) for 72 h. After harvest, all the plastic film and maize straw was removed.

### Soil CO_2_ emission, temperature, and water content

The CO_2_ emissions were measured using a GXH-3010E1 Portable Gas Analyser (Institute of Huayun Analytical Instrument Co. Ltd., Beijing, China) with custom-made polyvinyl closed soil respiration chambers (Xi’an Yangsheng Pipe Industry, China). The polyvinyl chambers (20 cm diameter and 15 cm height) were inserted firmly into the ground, up to a 5 cm depth, without removing any of the surface soil at least 48 hours prior to the first measurement. Two chambers were placed in each plot and remained at the same positions for the entire measurement period. For mulching treatment plots, chambers were inserted into the bare ground between two mulched strips (Fig. 4).

During the experiment, the daily mean air temperature, soil surface temperature, and rainfall were provided by the State Key Agro-Ecological Experimental Station, 1 km from the experiment site. For each plot, soil temperature and soil moisture were measured (depth of 5 cm) at four points near each soil respiration chamber. Soil temperature was measured using a hand-held soil thermometer (STP-1 Soil Temperature Probe, Institute of Huayun Analytical Instrument Co. Ltd.), and soil moisture was measured using a portable meter (TDR 300 Soil Moisture Meter, Spectrum Technologies Inc., Plainfield, IL, USA). The means of the soil temperature and soil moisture from the eight points (4 points per chamber × 2 chambers per plot) were used as the values for the plot.

In each year, starting at crop emergence, soil CO_2_ emission was measured every 7 to 10 days for each plot. In the event of rain, the CO_2_ emissions were measured at least 48 h after rainfall. The CO_2_ emission was measured as previously described^42^. Each measurement was taken between 09.00 and 11.00 h. The GXH-3010E1 Gas Analyser was attached to the data collector chambers with intake and outtake tubes (plastic material; length: 50 cm; inner diameter: 0.5 cm). At the time of data recording, the CO_2_ data (*X*_*1*_) were initially recorded without closing/covering the chamber, and then the chamber top was tightly closed with a cover equipped with a small fixed fan. The gas within the chamber was mixed for three minutes by this fan, and then the CO_2_ emission (*X*_*2*_) was recorded using the gas analyser. The CO_2_ emission rate was calculated using equation (1), as previously described^41^:


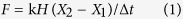


where *F* is the CO_2_ emission rate (g CO_2_ m^−2^ h^−1^), k is a constant with a value of 1.80 (25 °C), and *X*_*1*_ and *X*_*2*_ are the CO_2_ emission rates from the chambers before and after covering of the chambers, respectively. *H* is the height (m) of the chambers and Δ*t* is the time (h).

During the study period, cumulative soil surface CO_2_ emissions were calculated using equation (2)^43^:


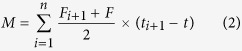


where, *M* is the cumulative emission of CO_2_-C (g CO_2_-C m^−2^), *F*_*i*_ is the first CO_2_ emission value (g CO_2_-C m^−2^ h^−1^) at time *t*_*i*_ (h), *F*_*i+1*_ is the same value at time *t*_*i+1*_ (h), and n is the total number of CO_2_ emission values.

To identify treatment combinations that can induce lower soil CO_2_ emission per unit grain yield, the amount of soil CO_2_ emission per unit grain yield was calculated using equation (3)^1^


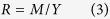


where *M* is the cumulative emission of CO_2_-C (t ha^−1^), and *Y* is the grain yield (t ha^−1^).

### Data analysis

For every block of each treatment, mean soil CO_2_ emissions, soil temperature, and soil moisture were calculated by averaging the three or more measurements in each sampling day. Data of each treatment were collected from the three blocks for analysis. Data in Tables 1 and 3 were statistically analysed by two-way ANOVA related to the split-plot experimental design. Data in Figs 2 and 3 and Table 4 were analysed by one-way ANOVA using least significant difference at P ≤ 0.05. The Pearson procedure of SPSS 16.0 was used to determine the correlation of soil CO_2_ emissions with soil temperature and moisture in Table 2. SAS software (version 8, SAS Institute, Cary, NC) was used for statistical analysis and Sigma Plot 10.0 (Aspire Software International, Ashburn, VA) was used for the illustrations.

## Additional Information

**How to cite this article**: Liu, Q. *et al*. Plastic-film mulching and urea types affect soil CO_2_ emissions and grain yield in spring maize on the Loess Plateau, China. *Sci. Rep.* **6**, 28150; doi: 10.1038/srep28150 (2016).

## Figures and Tables

**Table 1:** Effect of mulching (M), urea types (U) and there interactions (M × U) on soil CO_2_ emissions (g m^−2^ h^−1^) during the seedling, jointing, flare opening, blooming, filling, and milk stages of maize in 2013 and 2014.

	2013			2014		
	df	F value	Sig. (P)	df	F value	Sig. (P)
Seedling stage						
M	2	455.99	<0.0001^***^	2	21.29	0.0074^**^
U	1	2.97	0.1354	1	9.58	0.0212^*^
M × U	2	1.44	0.3085	2	30.63	0.0007^**^
Jointing stage						
M	2	22.88	0.0065^**^	2	29.86	0.0039^**^
U	1	3.40	0.1150	1	0.03	0.8688
M × U	2	0.45	0.6598	2	2.74	0.1426
Flare opening stage						
M	2	1356.15	<0.0001^***^	2	45.18	0.0018^**^
U	1	56.96	0.0003^**^	1	484.84	<0.0001^***^
M × U	2	16.51	0.0036^**^	2	258.42	<0.0001^***^
Blooming stage						
M	2	93.12	0.0004^**^	2	68.12	0.0008^**^
U	1	18.60	0.0050^**^	1	24.00	0.0027^**^
M × U	2	3.66	0.0915	2	5.18	0.0494^*^
Filling stage						
M	2	3.48	0.1333	2	24.75	0.0056^**^
U	1	3.84	0.0978	1	1.94	0.2129
M × U	2	0.26	0.7812	2	0.56	0.5976
Milk stage						
M	2	2.19	0.2282	2	14.99	0.0139^*^
U	1	5.67	0.0546	1	2.97	0.1353
M × U	2	6.39	0.0326^*^	2	0.59	0.5843

^*^P < 0.05, ^**^P < 0.01, ^***^P < 0.001.

**Table 2:** Correlations between soil CO_2_ emissions (F), soil temperature (T) and soil water content (W) at a 5-cm depth under NU, NC, TU, TC, BU and BC in 2013 and 2014.

Treatment	2013				2014			
	F - T		F - W		F - T		F - W	
	R^2^	P	R^2^	P	R^2^	P	R^2^	P
NU	0.124	0.603	0.121	0.612	0.011	0.896	0.425	0.100
NC	0.133	0.577	0.153	0.521	−0.119	0.662	0.501	0.048^*^
TU	0.231	0.327	0.12	0.614	0.139	0.608	−0.128	0.638
TC	0.373	0.106	0.165	0.486	0.115	0.671	0.127	0.640
BU	0.375	0.103	0.397	0.083	−0.073	0.787	0.128	0.637
BC	0.312	0.181	0.246	0.296	−0.026	0.923	0.075	0.782

^*^P < 0.05; R^2^, correlation coefficient; Number of samples was 20 in 2013 and 16 in 2014, respectively; NU, no plastic-film mulching with urea; NC, no plastic-film mulching with controlled release fertiliser (CRF); TU, transparent plastic-film mulching (PMt) with urea; TC, PMt with CRF; BU, black plastic-film mulching (PMb) with urea; BC, PMb with CRF.

**Table 3:** Effect of mulching (M), urea types (U) and there interactions (M × U) on cumulative soil CO_2_ emissions (t ha^−1^), grain yield (t ha^−1^), and cumulative soil CO_2_ emissions per unit grain yield during the two growing seasons.

	Soil CO_2_			Grain yield		Soil CO_2_ per yield	
	df	F value	Sig. (P)	F	Sig. (P)	F value	Sig. (P)
2013							
M	2	5531.01	<0.0001^***^	14.52	0.0010^**^	141.12	0.0002^**^
U	1	311.26	<0.0001^***^	25.07	0.0024^**^	46.5	0.0005^**^
M × U	2	42.71	0.0003^**^	16.42	0.0025^**^	17.05	0.0033^**^
2014							
M	2	9717.41	<0.0001^***^	105.23	0.0015^**^	129.52	0.0002^**^
U	1	1552.22	<0.0001^***^	55.42	0.0003^**^	1071.3	<0.0001^***^
M × U	2	14.21	0.0053^**^	153.91	0.0391^*^	10.92	0.0100^*^

*P < 0.05; **P < 0.01; ***P < 0.001.

**Table 4:** Cumulative soil CO_2_ emissions (t ha^−1^), grain yield (t ha^−1^), and cumulative soil CO_2_ emission per unit grain yield in the mulching and urea treatments during the two growing seasons.

Treatment	Soil CO_2_	Grain yield	Soil CO_2_ per yield
2013			
NU	19.24 ± 0.48 c	9.18 ± 0.19 c	2.10 ± 0.03 ab
NC	17.94 ± 0.30 d	9.04 ± 0.89 c	1.99 ± 0.12 bc
TU	23.65 ± 0.30 a	10.41 ± 1.01 bc	2.27 ± 0.13 a
TC	22.31 ± 0.50 b	12.35 ± 1.12 a	1.81 ± 0.10 c
BU	18.88 ± 0.34 c	11.80 ± 1.06 ab	1.60 ± 0.09 d
BC	18.63 ± 0.25 c	12.16 ± 1.23 a	1.54 ± 0.14 d
2014			
NU	29.72 ± 0.39 c	13.18 ± 1.35 d	2.26 ± 0.03 a
NC	25.86 ± 0.60 d	13.52 ± 1.39 cd	1.91 ± 0.01 bc
TU	34.55 ± 0.77 a	14.85 ± 1.47 bc	2.33 ± 0.07 a
TC	31.63 ± 0.40 b	16.02 ± 1.25 ab	1.98 ± 0.11 b
BU	29.32 ± 0.61 c	15.94 ± 1.16 ab	1.84 ± 0.06 c
BC	26.38 ± 0.57 d	16.64 ± 1.49 a	1.59 ± 0.09 d

Data presented are means ± s.e.m. For each season, numbers followed by different letters within a measurement are significantly different at P ≤ 0.05 (least significant difference test). NU, no plastic-film mulching with urea; NC, no plastic-film mulching with controlled release fertiliser (CRF); TU, transparent plastic-film mulching (PMt) with urea; TC, PMt with CRF; BU, black plastic-film mulching (PMb) with urea; BC, PMb with CRF.
